# Cognitive, Behavioral, and Emotional Manifestations in Nodding Syndrome: A Neuropsychological Narrative Review

**DOI:** 10.31083/RN47379

**Published:** 2026-02-26

**Authors:** Ana Cristina de Castro, María J. García-Rubio

**Affiliations:** ^1^Department of Nursing, Faculty of Medicine and Health Sciences, Universidad de Alcalá, 28801 Madrid, Spain; ^2^Global Neuroscience and Social Change, Universidad Internacional de Valencia, 46002 Valencia, Spain; ^3^Cognition, Affect and Resilience (CARE) Research Group, Health Sciences Faculty, Universidad Internacional de Valencia, 46002 Valencia, Spain

**Keywords:** nodding syndrome, neuropsychology, cognitive dysfunction, epilepsy, Africa South of the Sahara

## Abstract

**Background:**

Nodding syndrome is a childhood-onset epileptic encephalopathy described in onchocerciasis-endemic regions of sub-Saharan Africa. Although characterized by recurrent atonic seizures with repetitive head-nodding movements, increasing evidence suggests that the condition extends beyond a purely motor epilepsy and involves progressive cognitive, behavioral, and emotional impairment.

**Methods:**

This study was designed as a narrative review with a specific neuropsychological focus. A literature search was conducted in PubMed, Scopus, and Google Scholar for publications between 2013 and 2025. Studies describing cognitive, behavioral, emotional, psychiatric, neuroimaging, or neuropathological findings in patients with nodding syndrome were included. Due to heterogeneity in study design and assessment methods, findings were synthesized narratively.

**Results:**

Across studies, nodding syndrome is consistently associated with progressive cognitive decline affecting attention, processing speed, executive functions, memory, and global intellectual functioning. Behavioral disturbances such as irritability, aggression, and emotional lability are frequently reported, alongside depressive symptoms and social withdrawal. Neuroimaging findings commonly demonstrate cortical and cerebellar atrophy, while neuropathological studies report tau-protein deposition and neuronal loss, supporting diffuse cerebral involvement with possible neurodegenerative features.

**Conclusions:**

Nodding syndrome represents a complex epileptic encephalopathy characterized not only by seizures but also by significant cognitive, behavioral, and emotional impairment. A clearer neuropsychological characterization may inform clinical assessment and guide future research aimed at improving supportive and rehabilitative interventions.

## 1. Introduction

Nodding syndrome is a rare childhood-onset epileptic encephalopathy that was first described in the early 2000s in rural communities of East and Central Africa [1]. It is characterized by recurrent atonic seizures with repetitive forward head-nodding movements. Nodding syndrome affects previously healthy children, often leading to a progressive neurological syndrome rather than an isolated epileptic condition.

Although thousands of cases have been reported in Uganda, South Sudan, and Tanzania, as well as sporadically in Cameroon and the Democratic Republic of Congo, the etiology remains unclear. Epidemiological associations with onchocerciasis and emerging neuropathological evidence of tau protein deposition suggest that nodding syndrome may represent a complex epileptic encephalopathy with neurodegenerative features rather than a purely functional seizure disorder [2, 3].

Current treatment is symptomatic, focusing on seizure control and nutritional support, but with limited efficacy and no disease-modifying effects [4]. While these clinical aspects are essential for contextualization, increasing evidence indicates that the long-term burden of the syndrome is largely driven by cognitive deterioration and behavioral and emotional disturbances, which remain insufficiently characterized.

From a neuropsychological perspective, nodding syndrome poses a major challenge, as much of the associated disability arises from progressive impairments in cognition, behavior, and emotional regulation rather than from seizures alone. Despite this, systematic neuropsychological assessments and interventions have received little attention in the scientific literature, with available data limited to isolated observations or indirect descriptions within broader clinical studies.

This gap has been highlighted by recent scoping and methodological reviews, which emphasize the fragmentation of existing evidence and the lack of integrative frameworks addressing neurocognitive and behavioral functioning in nodding syndrome. In this context, de Castro and Ortega-Deballon [5] underscored the scarcity of studies that specifically examine cognitive and behavioral outcomes and called for approaches capable of synthesizing clinical, epidemiological, and psychosocial findings within a coherent model. These observations strongly support the relevance of a focused neuropsychological review.

The objective of this narrative review is to synthesize and critically analyze the available evidence on the cognitive, behavioral, and emotional manifestations of nodding syndrome, with particular emphasis on the neuropsychological domains most consistently affected, the assessment methods employed, and the functional impact on patients and their families. By adopting this perspective, the review aims to move beyond descriptive clinical accounts and contribute to a clearer characterization of the neuropsychological profile of the disorder, identifying current limitations and directions for future research and intervention.

## 2. Methodology

A comprehensive literature search was conducted to identify relevant publications on nodding syndrome, with particular attention to its cognitive, behavioral, and emotional manifestations. This study was designed as a narrative review with a specific neuropsychological focus. Between July and August 2025, the PubMed (https://pubmed.ncbi.nlm.nih.gov/), Scopus (https://www.scopus.com/), and Google Scholar (https://scholar.google.com/) databases were searched. The search was restricted to literature published between 2013 and 2025 to prioritize studies providing more detailed and systematic descriptions of cognitive, behavioral, and emotional functioning. Earlier seminal publications were used to provide contextual background but were not included in the formal synthesis due to their limited neuropsychological characterization.

Combinations of English and Spanish keywords were used, including: “nodding syndrome”, “síndrome de cabeceo”, “cognitive impairment”, “behavioral symptoms”, “psychiatric”, “neuropsychology”, “Uganda”, and “Onchocerca-associated epilepsy”.

**Fig. 1.**
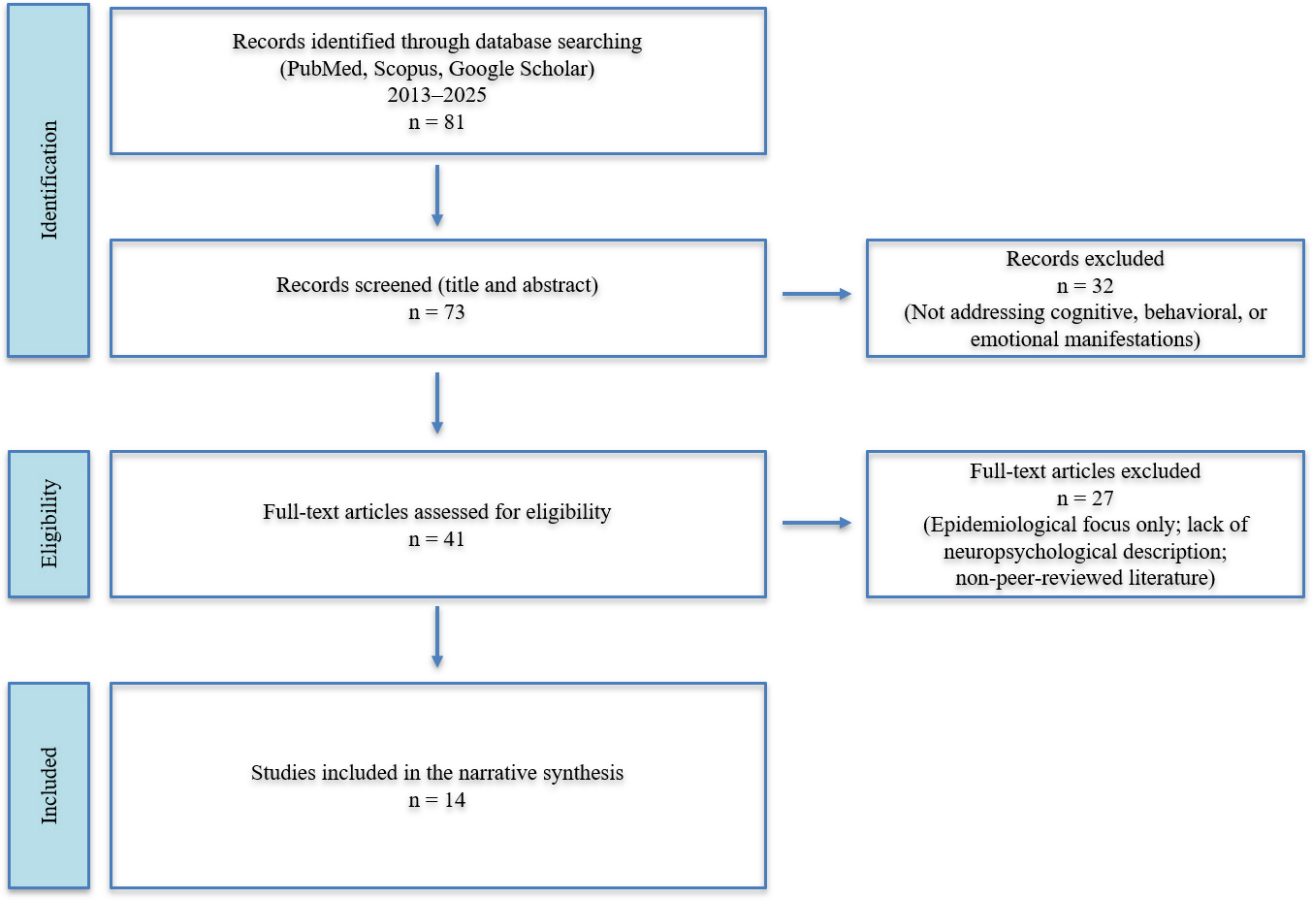
**Study selection flow diagram (PRISMA-style)**. PRISMA, preferred reporting items for systematic reviews and meta-analyses.

The initial search yielded approximately 81 unique references. After removing duplicates and screening titles and abstracts, 73 potentially relevant publications were identified. Of these, 41 articles were assessed in full text, and 14 studies met the inclusion criteria and were included in the narrative synthesis. The study selection process is summarized in Fig. 1.

Studies were considered eligible if they involved patients with a confirmed clinical diagnosis of nodding syndrome or neuropathological analyses attributable to this condition, and if they explicitly described cognitive, behavioral, emotional, or psychiatric manifestations. Observational studies (cross-sectional studies, cohort studies, or case series), neuropathological reports, and narrative reviews containing original clinical descriptions were included. Studies focusing exclusively on epidemiological, environmental, or genetic aspects without a clinical description of cognitive or psychiatric features, grey literature lacking peer review, and general reviews with no analysis of specific clinical manifestations were excluded.

From each included publication, data were extracted regarding author and year, country or region, study design, number of patients, and the neuropsychological or psychiatric assessment methods employed, as well as the main cognitive, behavioral, and emotional findings. These data are summarized in the corresponding tables.

Given the narrative nature of this review, no formal meta-analysis or standardized risk-of-bias assessment was performed. Nevertheless, it should be noted that most included studies were observational case series or small cohort studies, which are inherently associated with a high risk of selection bias, assessment method heterogeneity, and limited generalizability. These methodological limitations were considered when synthesizing and interpreting the findings across the studies. When standardized neuropsychological batteries were not reported, cognitive, behavioral, and emotional domains were inferred from detailed clinical descriptions, functional outcomes, and caregiver reports, in line with narrative syntheses of rare neurological disorders and low-resource settings.

## 3. Results

The studies included in this narrative review were analyzed to identify convergent patterns and sources of variability in the clinical and neuropsychological manifestations of nodding syndrome. Table 1 (Ref. [2, 6, 7, 8, 9, 10, 11, 12, 13, 14, 15, 16, 17, 18]) summarizes the main methodological characteristics of the 14 reviewed studies, including study design, populations, and assessment approaches. Table 2 (Ref. [6, 7, 8, 9, 10, 11, 12, 13, 14, 16]) provides an integrative synthesis of neuropsychological findings organized by cognitive, behavioral, and emotional domains, allowing comparison across studies beyond individual descriptions.

**Table 1.** **Characteristics of the studies included in the narrative review**.

Study [ref]	Country/Region	Study design	N (patients or studies)	Assessment methods	Data type
Dowell *et al*. [6]	Uganda, South Sudan	Clinical and epidemiological description	>100	Community clinical assessment	Clinical
Idro *et al*. [7]	Uganda	Case series	62	Clinical evaluation, MRI, caregiver interviews	Clinical, neuroimaging
de Polo *et al*. [15]	South Sudan	Case series	21	EEG, clinical interviews	EEG, clinical
Colebunders *et al*. [17]	Uganda	Hypothesis/narrative review	n/a	Clinical and epidemiological synthesis	Review
Idro *et al*. [2]	Uganda	Review/hypothesis	n/a	Clinical and epidemiological synthesis	Review
Stacey *et al*. [11]	Multiregional	Systematic review and meta-analysis	12 studies	Quantitative synthesis	Review
Olum *et al*. [12]	Multiregional	Concise narrative review	n/a	Narrative synthesis	Review
Abd-Elfarag *et al*. [13]	Multiregional	Scoping review	34 studies	Narrative literature analysis	Review
Morin *et al*. [10]	Cameroon	Cross-sectional clinical study	47	Structured neuropsychological testing	Neuropsychological
Mazumder *et al*. [14]	Uganda	Comparative neuroimaging study	30 NS/epilepsy controls	Volumetric MRI	Neuroimaging
Abd-Elfarag *et al*. [8]	South Sudan (Mundri)	Community-based cross-sectional study	22 confirmed/22 suspected	Structured interviews, functional surveys	Clinical, behavioral
Pollanen *et al*. [16]	Uganda	Neuropathological study	8 post-mortem brains	Histopathological analysis	Neuropathology
Kegele *et al*. [9]	Uganda	Longitudinal clinical cohort	>100	Serial clinical evaluations	Clinical
Spencer *et al*. [18]	Multiregional	Experimental hypothesis	n/a	Environmental and molecular hypothesis	Hypothesis

**Note**. NS, nodding syndrome; EEG, electroencephalogram; MRI, magnetic resonance imaging. Table 1 summarizes methodological characteristics and assessment approaches of the 14 studies included in the narrative synthesis. n/a, not applicable.

**Table 2.** **Synthesis of neuropsychological findings in nodding syndrome by domain**.

Domain	Main findings	Assessment methods	Studies reporting findings [ref]	Consistency across studies
Attention/processing speed	Reduced sustained attention, slowed information processing	Clinical observation, structured testing	[6, 7, 8, 9, 10]	High
Executive functions	Impaired planning, inhibitory control, cognitive flexibility	Standardized neuropsychological tests; clinical assessment	[7, 9, 10]	Moderate–High
Memory and learning	Difficulties in new learning and memory retention	Caregiver reports; standardized testing	[8, 9, 10]	Moderate
Global cognition/intellectual regression	Progressive intellectual decline and developmental regression	Clinical evaluation; longitudinal follow-up	[6, 7, 9]	High
Psychomotor functioning	Psychomotor slowing and reduced initiation	Clinical observation	[6, 9]	Moderate
Behavioral dysregulation	Irritability, aggression, impulsivity, emotional lability	Caregiver interviews; community studies	[8, 11, 12, 13]	High
Emotional symptoms	Depressive symptoms, social withdrawal, apathy	Clinical interviews; caregiver reports	[11, 12, 13]	Moderate
Functional impact	Impaired academic performance, reduced autonomy, social stigma	Functional and community-based assessments	[6, 8, 9]	High
Neuroimaging correlates	Cortical and cerebellar atrophy associated with cognitive impairment	MRI	[7, 14]	High
Neuropathological correlates	Tau-protein deposition, neuronal loss, cortical gliosis	Post-mortem histopathology	[16]	Moderate

**Note**. Consistency reflects qualitative convergence of findings across studies rather than a formal quantitative assessment. Assignment of neuropsychological domains was based on explicit test results when available and, more frequently, on clinical descriptions, functional impairment, and caregiver reports, due to the limited use of standardized neuropsychological batteries across studies.

### 3.1 Cognitive Impairment and Neuropsychological Profile

Across studies, cognitive impairment emerges as a central and persistent feature of nodding syndrome, extending beyond the effects of epileptic seizures alone. Clinical, cross-sectional, and longitudinal investigations consistently report deficits in attention, memory, learning abilities, and global intellectual functioning, with clear repercussions on academic performance, daily functioning, and autonomy [6, 7, 8, 9].

Psychomotor slowing and intellectual regression are frequently described, particularly in patients with longer disease duration. These impairments seem to persist even when seizures are partially stabilized with antiepileptic treatment [8, 9]. As synthesized in Table 2, these cognitive deficits show a high degree of consistency across studies despite the heterogeneity in their assessment methods.

A key contribution to the neuropsychological characterization of the disorder is provided by Morin *et al*. [10], who incorporated structured neuropsychological testing in patients with epilepsy associated with Onchocerca volvulus. Unlike most studies relying on clinical observation or caregiver reports, this work employed standardized measures of attention, memory, and executive functions, demonstrating a generalized cognitive impairment with prominent involvement of executive and attentional processes [10]. This pattern supports the presence of diffuse cortical and subcortical dysfunction, consistent with the neuroimaging and neuropathological findings summarized in Table 2.

### 3.2 Behavioral and Emotional Disturbances

Behavioral and emotional symptoms are consistently reported across community-based studies, clinical series, and narrative reviews, constituting a major source of functional and social disability. Common manifestations include irritability, aggression, impulsivity, emotional lability, depressive symptoms, and social withdrawal, which profoundly affect family dynamics and community integration [11, 12, 13].

As shown in Table 2, these disturbances are described across diverse settings and age groups, although assessment methods are often limited to caregiver reports or unstructured clinical interviews. Contextual factors such as social stigma, poverty, and limited access to healthcare further exacerbate these symptoms, contributing to the chronicity and severity of disability [8].

### 3.3 Paraclinical and Neuropathological Correlates

Paraclinical findings provide convergent evidence of the diffuse cerebral involvement underlying the observed neuropsychological profile. Neuroimaging studies consistently demonstrate cortical and cerebellar atrophy, which correlates with longer disease duration and greater cognitive impairment [7, 14]. Electroencephalographic recordings typically reveal diffuse background slowing and generalized epileptiform discharges, reflecting both the epileptic nature of the syndrome and its association with cognitive progression [15].

Neuropathological investigations further support a neurodegenerative component. Post-mortem examinations have identified abnormal tau-protein deposits, neuronal loss, and cortical gliosis, reinforcing the hypothesis that nodding syndrome may involve a secondary tauopathy contributing to progressive cognitive decline [16].

### 3.4 Etiological Considerations in Relation to Neuropsychological Outcomes

Most reviewed studies support an association between nodding syndrome and onchocerciasis, proposing immunoinflammatory mechanisms involving Onchocerca volvulus and its endosymbiont Wolbachia as potential contributors to brain injury [2, 17]. While these hypotheses provide important context, direct causal links between infection and specific neuropsychological deficits remain unproven.

More recent hypothesis-driven research has suggested a possible contribution of environmental factors and biotoxins capable of altering gene regulation, including pathways involving methyl-CpG-binding protein 2 (*MECP2*), which may influence cognitive and behavioral functioning [18]. These proposals remain exploratory but may help explain the heterogeneity of neuropsychological outcomes summarized in Table 2.

### 3.5 Integrative Synthesis

Despite heterogeneity in study designs, sample sizes, and assessment methods, there is strong convergence across the reviewed literature that nodding syndrome is characterized by the combination of epileptic seizures, progressive cognitive decline, and significant behavioral and emotional disturbances. Systematic, scoping, and narrative reviews consistently emphasize that cognitive and behavioral impairments represent the primary drivers of long-term disability, underscoring the relevance of a focused neuropsychological perspective [11, 12, 13].

## 4. Discussion

The findings of this review highlight that nodding syndrome is a neurological entity that extends beyond the motor phenomenon of epileptic seizures and should be understood as a progressive epileptic encephalopathy. Importantly, the present synthesis emphasizes that cognitive, behavioral, and emotional impairments represent core features of the disorder rather than secondary consequences of seizure activity alone. Clinical and epidemiological studies consistently show that patients experience persistent cognitive deterioration accompanied by behavioral and emotional disturbances, resulting in a disability burden far greater than that attributable to seizures alone [7, 8, 9]. From a neuropsychological perspective, this pattern is consistent with a diffuse and progressive disruption of higher-order cognitive and regulatory functions.

A neuroimaging and neuropathological study provides additional evidence of diffuse and progressive cerebral involvement, with cortical and cerebellar atrophy, electroencephalographic slowing, and abnormal tau-protein deposits [16]. These findings offer a plausible structural substrate for the observed neuropsychological profile, characterized by attentional deficits, psychomotor slowing, executive dysfunction, and impaired emotional regulation. The persistence of cognitive impairment even in patients with partial symptomatic seizure control further supports the notion that neurocognitive decline follows a trajectory that is at least partially independent of seizure frequency [9].

The etiology of nodding syndrome remains a matter of debate. The most widely supported hypothesis continues to be the epidemiological association with onchocerciasis, based on field studies and immunological analyses suggesting a neuroinflammatory mechanism mediated by the host response to Onchocerca volvulus and its bacterial endosymbiont Wolbachia [2, 17]. While etiological considerations provide important context, the present review focuses primarily on how these mechanisms may converge on neuropsychological outcomes rather than on causation per se. In parallel, recent research has proposed the contribution of environmental factors or biotoxins capable of altering gene expression in neurodevelopmental pathways [18]. These hypotheses remain exploratory and underline the complexity of linking biological mechanisms to specific cognitive and behavioral phenotypes.

Beyond cognitive decline, patients with nodding syndrome frequently exhibit significant emotional and behavioral disturbances. Several studies describe irritability, social withdrawal, depressive symptoms, and aggressive behavior, whose severity tends to increase as the disease progresses [11, 12, 13]. Together with cognitive deterioration, these symptoms define a distinctive neuropsychological profile in which deficits in attention, executive control, and emotional self-regulation interact with adverse psychosocial contexts, amplifying functional disability. Despite their relevance, behavioral and emotional manifestations are still infrequently assessed using standardized instruments, limiting their characterization and representing a critical gap in the existing literature.

Regarding therapeutic approaches, current data confirm that available interventions are primarily symptomatic and offer limited benefit. Antiepileptic treatment may reduce seizure frequency in some cases, yet cognitive and behavioral deterioration often persists, leading to chronic disability [5, 9]. From a neuropsychological standpoint, these findings underscore the need to complement seizure control with targeted cognitive rehabilitation, educational adaptations, and psychosocial interventions, particularly in pediatric populations.

This review has several limitations that must be acknowledged. Most included studies feature small sample sizes, observational designs, and limited standardization of neuropsychological assessments. In many cases, cognitive and behavioral domains were inferred from clinical descriptions, functional outcomes, and caregiver reports rather than from comprehensive neuropsychological batteries. Nevertheless, the convergence of findings across diverse geographic and methodological contexts strengthens the validity of the synthesized neuropsychological patterns.

Taken together, the available evidence indicates that nodding syndrome represents an epilepsy with a high burden of cognitive, behavioral, and emotional disability, probably of multifactorial origin and with a neurodegenerative component. These impairments constitute the main drivers of long-term functional limitation and social burden, reinforcing the importance of a neuropsychological framework for both research and clinical management.

## 5. Conclusions

Nodding syndrome is a severe and progressive childhood-onset epileptic encephalopathy characterized not only by recurrent atonic seizures but also by sustained cognitive decline and behavioral and emotional disturbances that lead to chronic disability. The present review highlights that neuropsychological impairment is a central feature of the syndrome and a key determinant of its functional and social impact.

Convergent neuroimaging and neuropathological findings support the presence of diffuse brain involvement with a possible neurodegenerative component, while current etiological hypotheses point to a complex interaction between Onchocerca volvulus infections, secondary immunoinflammatory mechanisms, and potential environmental or epigenetic cofactors. Understanding how these mechanisms translate into specific neuropsychological profiles remains a major challenge for future research.

In the absence of disease-modifying therapies, there is a pressing need for interdisciplinary management strategies simultaneously addressing seizure control, neuropsychological assessment, cognitive rehabilitation, and psycho-emotional support. Likewise, longitudinal studies incorporating standardized neuropsychological measures are essential to clarify disease progression and evaluate the impact of targeted interventions on cognitive and behavioral outcomes.

## Availability of Data and Materials

Not applicable, as this study is a narrative review and does not involve original data collection or generated datasets.

## References

[1] Winkler AS, Friedrich K, König R, Meindl M, Helbok R, Unterberger I (2008). The head nodding syndrome-clinical classification and possible causes. *Epilepsia*.

[2] Idro R, Opar B, Wamala J, Abbo C, Onzivua S, Mwaka AD (2016). Nodding syndrome in Ugandan children-clinical features, brain imaging, and complications: a case series. *BMJ Open*.

[3] Ridler C (2018). Nodding syndrome discovered to be a tauopathy. *Nature Reviews. Neurology*.

[4] Idro R, Musubire KA, Byamah Mutamba B, Namusoke H, Muron J, Abbo C (2013). Proposed guidelines for the management of nodding syndrome. *African Health Sciences*.

[5] De Castro AC, Ortega-Deballon I (2020). Nodding syndrome: bridging the gap-a scoping review protocol. *BMJ Open*.

[6] Dowell SF, Sejvar JJ, Riek L, Vandemaele KAH, Lamunu M, Kuesel AC (2013). Nodding syndrome. *Emerging Infectious Diseases*.

[7] Idro R, Namusoke H, Abbo C, Mutamba BB, Kakooza-Mwesige A, Opoka RO (2014). Patients with nodding syndrome in Uganda improve with symptomatic treatment: a cross-sectional study. *BMJ Open*.

[8] Abd-Elfarag GOE, Edridge AWD, Spijker R, Sebit MB, van Hensbroek MB (2021). Nodding Syndrome: A Scoping Review. *Tropical Medicine and Infectious Disease*.

[9] Kegele J, Wagner T, Kowenski T, Wiesmayr M, Gatterer C, Alber M (2024). Long-term clinical course and treatment outcomes of individuals with Nodding Syndrome. *Journal of the Neurological Sciences*.

[10] Morin A, Guillaume M, Ngarka L, Tatah GY, Siewe Fodjo JN, Wyart G (2021). Epilepsy in the Sanaga-Mbam valley, an onchocerciasis-endemic region in Cameroon: electroclinical and neuropsychological findings. *Epilepsia Open*.

[11] Stacey HJ, Woodhouse L, Welburn SC, Jones JD (2019). Aetiologies and therapies of nodding syndrome: a systematic review and meta-analysis. *Journal of Global Health Reports*.

[12] Olum S, Scolding P, Hardy C, Obol J, Scolding NJ (2020). Nodding syndrome: a concise review. *Brain Communications*.

[13] Abd-Elfarag GOE, Mathewson JD, Emmanuel L, Edridge AWD, van Beers S, Sebit MB (2023). Nodding Syndrome: Clinical Characteristics, Risks Factors, Access to Treatment, and Perceptions in the Greater Mundri Area, South Sudan. *Pathogens (Basel, Switzerland)*.

[14] Mazumder R, Lubowa SK, Salamon N, Jackson NJ, Kawooya M, Akun PR (2022). Comparison of Structural Changes in Nodding Syndrome and Other Epilepsies Associated With Onchocerca volvulus. *Neurology(R) Neuroimmunology & Neuroinflammation*.

[15] de Polo G, Romaniello R, Otim A, Benjamin K, Bonanni P, Borgatti R (2015). Neurophysiological and clinical findings on Nodding Syndrome in 21 South Sudanese children and a review of the literature. *Seizure*.

[16] Pollanen MS, Onzivua S, McKeever PM, Robertson J, Mackenzie IR, Kovacs GG (2023). The spectrum of disease and tau pathology of nodding syndrome in Uganda. *Brain: a Journal of Neurology*.

[17] Colebunders R, Hendy A, Nanyunja M, Wamala JF, van Oijen M (2014). Nodding syndrome-a new hypothesis and new direction for research. *International Journal of Infectious Diseases: IJID: Official Publication of the International Society for Infectious Diseases*.

[18] Spencer PS, Valdes Angues R, Palmer VS (2024). Nodding syndrome: A role for environmental biotoxins that dysregulate MECP2 expression?. *Journal of the Neurological Sciences*.

